# Effect of Selected Entomopathogenic Fungal Species on Embryonic Development of *Ascaris suum* (Nematoda)

**DOI:** 10.3390/ani13243782

**Published:** 2023-12-08

**Authors:** Kinga Mazurkiewicz-Zapałowicz, Bogumiła Pilarczyk, Lidia Kołodziejczyk, Cezary Tkaczuk, Magdalena Twarużek, Łukasz Łopusiewicz, Jan Grajewski, Ewa Dzika, Elżbieta Kalisińska

**Affiliations:** 1 Department of Hydrobiology, Ichthyology and Biotechnology of Reproduction, West Pomeranian University of Technology in Szczecin, Kazimierza Królewicza 4, 71-550 Szczecin, Poland; kmazurkiewicz@zut.edu.pl; 2 Department of Animal Reproduction Biotechnology and Environmental Hygiene, West Pomeranian University of Technology in Szczecin, Janickiego 29, 71-270 Szczecin, Poland; bogumila.pilarczyk@zut.edu.pl; 3 Department of Biology and Medical Parasitology, Pomeranian Medical University, al. Powstańców Wielkopolskich 72, 70-111 Szczecin, Poland; lkolo@pum.edu.pl (L.K.); elzbieta.kalisinska@pum.edu.pl (E.K.); 4 Institute of Agriculture and Horticulture, University in Siedlce, Prusa 14, 08-110 Siedlce, Poland; cezary.tkaczuk@uph.edu.pl; 5 Department of Physiology and Toxicology, Kazimierz Wielki University, Chodkiewicza 30, 85-064 Bydgoszcz, Poland; twarmag@ukw.edu.pl (M.T.); jangra@ukw.edu.pl (J.G.); 6 Center of Bioimmobilisation and Innovative Packaging Materials, West Pomeranian University of Technology in Szczecin, Janickiego 35, 71-270 Szczecin, Poland; 7 Department of Medical Biology, University of Warmia and Mazury, Żołnierska 14c, 10-561 Olsztyn, Poland; e.dzika@uwm.edu.pl

**Keywords:** *Ascaris suum*, ovistatic effect, entomopathogenic fungi, biocontrol

## Abstract

**Simple Summary:**

Endoparasites such as *Ascaris suum* can pose a serious threat to the health of livestock and, consequently, humans. One promising way of controlling the threat is the use of natural enemies/antagonistic fungi. In this study, we examined the effects of entomopathogenic fungi (naturally attacking insects) as a bioregulator in the invasive stages of the parasitic nematode *A. suum*. The conducted study indicates that none of the fungal strains tested have nematocidal activity against *A. suum* eggs, and they do not meet the criteria required for use in the bioregulation of the parasite’s dispersal stages. Among the strains tested, *Isaria fumosorosea* and *Metarhizium robertsii* stood out, combining the highest metabolic activity with nematocidal activity against *A. suum*.

**Abstract:**

The aim of the study was to evaluate the potential of using five selected species of entomopathogenic fungi (*Beauveria bassiana*, *B. brongniartii*, *Conidiobolus coronatus*, *Isaria fumosorosea*, and *Metarhizium robertsii*) in the bioregulation of the dispersive stages of the parasitic nematode—*Ascaris suum*. Experimental cultures of each of the selected entomopathogenic fungi, as well as a control culture without fungi, were incubated with *A. suum* eggs at 26 °C for 28 days. Development of the *A. suum* eggs was observed using a light microscope on the 7th, 14th, 21st, and 28th days of incubation. The API-ZYM^®^ test was used to determine, semiquantitatively, the activity of 19 hydrolytic enzymes from the entomopathogenic fungi. The cytotoxicity of the fungi was determined using tetrazole salt MTT. It was found that none of the five tested strains of entomopathogenic fungi showed an ovicidal effect, and none of them colonized the *A. suum* egg shells. However, ovistatic activity was observed mainly until the 14th day of incubation by *I. fumosorosea*, *M. robertsii*, and *B. bassiana*. In the MTT test, *M. robertsii* showed moderate cytotoxicity, while the other species showed low cytotoxicity. Among the strains tested, *I. fumosorosea* showed the highest spectrum of hydrolase production (13 out of 19 enzymes gave a positive reaction from 3 to 5; 20–40 nM or more). The absence of morphological changes in the *A. suum* egg shells suggests that the antagonistic effect of the studied entomopathogenic fungi may be due to their cytotoxicity, associated with the production of secondary metabolites—toxins (*M. robertsii*) and enzymatic activity (*I. fumosorosea*).

## 1. Introduction

As a result of raising and breeding pigs, a large amount of organic matter is introduced into the environment in the form of animal feces. This leads to the entry of various forms of parasites into the soil. The use of manure from pig farms poses potential risks to human and animal health due to soil-transmitted helminths, such as *Ascaris* spp. [1]. Their eggs can contaminate soils and surface waters, may be transported to wastewater treatment plants, and may be deposited in sludge that is sometimes used as an organic fertilizer [1,2,3,4].

The very high reproductive potential of *Ascaris* (200,000 eggs/day), the longevity of the invasive eggs, and their resistance to the influence of harmful external factors pose a significant problem in the control of ascariasis, while at the same time resulting in a high extensiveness of infection in humans and pigs [5,6,7]. The prevalence of *A. suum* ranges from a dozen to more than 60% by country, population, husbandry, diet, and age of the carrier pigs [8,9,10,11]. The extensiveness of *A. suum* infection in pigs in Poland is 30–60% [7,8,12] and is also high in other countries. For instance, it ranges from 15–64% in Denmark [10] and is 32.59% in Mumbai, India [9], 25.9% in Ethiopia [13], and 44.5% in South Africa [11]. Pigs living outdoors (including organic farms) are at higher risk of *Ascaris* infection than pigs housed indoors [10,14,15]. Ascariasis in pigs is a major economic problem due to detrimental carcass composition, reduced feed conversion efficiency, and losses to the meat industry, but it is also a public health threat [3,14,16,17].

According to Stewart [18], losses in the United States due to swine liver seizures (the effect of *A. suum* infestation) are estimated at USD 17.5 million per year, and losses associated with reduced feed conversion by animals are an additional USD 60.1 million per year. Some studies have shown that cross-species transmission occurs between pigs and humans living in close proximity or where pig manure is used as fertilizer on vegetables for human consumption. For this reason, *A. suum* is considered a zoonotic pathogen [14,16,19]. Furthermore, a number of researchers have suggested that *A. suum* and *A. lumbricoides* are a single species that is only reproductively isolated [20,21,22].

Eggs of the soil-transmitted parasitic nematodes *Ascaris*, *Trichuris*, and *Toxocara* have been considered as indicators in assessing the hygienic status of sewage sludge and organic fertilizers. The eggs of these parasites have been shown to be more resistant to various sanitation methods (liming, pasteurization, composting) than other animal and human endoparasites [2,4,15,23]. Due to the economic and health significance of nematode infections (especially ascariasis), as well as some regulations concerning organic farms (especially in Europe where the use of chemical anthelmintics is often prohibited), investigations on natural means active against human and animal parasitic nematodes have been conducted for years [1,24].

One of the methods used to tackle parasitic infestations in humans and animals are fungi, which possess various abilities to reduce helminth populations. Some fungi, such as entomopathogenic fungi, enter the host (insect) through the cuticle and initiate the development of infection, which then occurs in three main phases. The first phase is associated with the adhesion of conidial spores to the host body and their germination in the epicuticle. The second phase involves penetration of the cuticle by mycelial hyphae. This process, according to Mustafa and Kaur [25], is the result of mechanical forces together with enzymes produced by the fungi (proteases, chitinases, lipases, and lipoxygenases). The final stage is the complete destruction of the host and its death. In the course of zoomycosis, fungal metabolites are particularly active, including depsipeptides (e.g., Dsx destructins) produced by members of the genus *Metarhizium* [26] and *Conidiobolus* [27], which cause convulsions, loss of motor coordination, and paralysis in insects [28]. The nematostatic and nematocidal effects of some insecticidal fungi in reducing helminth populations that cause parasitosis in humans and animals are also known. The ovicidal effects of *Pochonia chlamydosporia* (H.C. Evans) have been reported in nematode species such as *Trichuris vulpis* [29], *Toxocara vitulorum* [30], *A. suum* [31], and the tapeworm *Taenia saginata* [32]. Penetration of *P. chlamydosporia* mycelia has also been observed in eggs of the flukes *Schistosoma mansonii* [33] and *Fasciola hepatica* [34]. Entomopathogenic fungi have also shown inhibitory effects on the development of *A. suum* and *A. lumbricoides* eggs. Of note among these species are the following strains: *Paecilomyces variotii* and *P. viridis* [35], *Isaria fumosorosea* (=*Paecilomyces fumosoroseus*), *P. lilacinus*, *Metarhizium flavoviride*, *M. anisopliae* [36,37], *Metacordyceps chlamydosporia* (=*Verticillium chlamydosporium*), and *Pochonia chlamydosporia* [31,38,39,40,41].

It seems reasonable to learn about the interaction of other species of entomopathogenic fungi with the dispersal stages of parasitic geohelminths. Therefore, the aim of this study was to evaluate the potential of selected entomopathogenic fungal species (*Beauveria bassiana*, *B. brongniartii*, *Conidiobolus coronatus*, *Isaria fumosorosea*, and *Metarhizium robertsii*) for use in the bioregulation of the invasive stages of the parasitic nematode *Ascaris suum*.

## 2. Materials and Methods

### 2.1. Fungi

**Figure 1:**
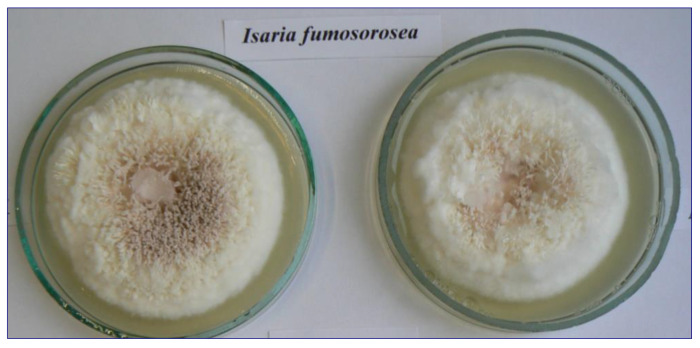
Strain of *Isaria fumosorosea* (photo by C. Tkaczuk).

**Figure 2:**
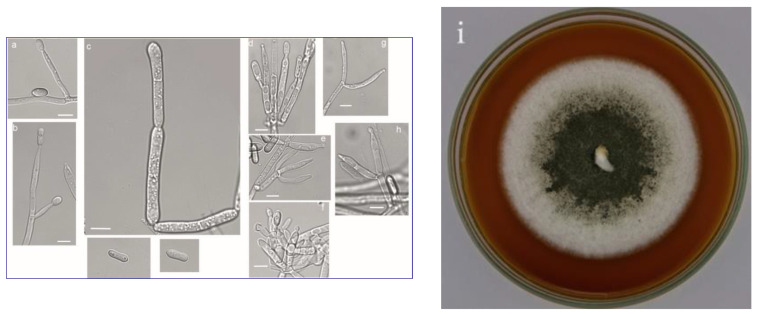
*Metarhizium robertsii*; (**a**–**h**) conidiophore and conidia, white scale bar—5 µm; (**i**) colony on agar medium (photo by C. Tkaczuk and S. Różalska).

Strains of five species of entomopathogenic fungi were selected for the study: *Beauveria bassiana* (Bals.-Criv.) Vuill., *B. brongniartii* (Sacc.) Petch, *Conidiobolus coronatus* (Costantin) A. Batko, *Isaria fumosorosea* Wize, and *Metarhizium robertsii* (J.F. Bisch.), S.A. Rehner and Humber. Isolates of these entomopathogenic fungi were obtained from the collection of the Department of Horticulture and Plant Protection at the University in Siedlce. Fungal strains of *Beauveria bassiana* (UPH 34), *Isaria fumosorosea* (UPH 42) (Figure 1), and *Metarhizium robertsii* (UPH 21) (Figure 2) were obtained from soils in cultivated fields near Siedlce (Masovian Voivodeship) using the *Galleria* bait method [42]. Isolates of the fungi *Beauveria brongniartii* (UPH 66) and *Conidiobolus coronatus* (UPH 50) were also isolated from cultivated soil, but using a selective medium [43]. The nomenclature of the fungi is based on the Index Fungorum (http://www.indexfungorum.org accessed on 20 October 2023). Single-spore cultures of the strains were grown on PDA medium (Merck). Prior to the experiment, the fungi were grown on Sabouraud medium (SDA) and stored at 4 °C. 

Discs (ø 4 mm) were cut from the 3-week-old mycelium of these cultures and placed on PDA medium. Incubation was carried out in the dark at 25 °C for 21 days, after which new discs (ø 4 mm) were cut and transferred to Petri dishes (ø 50 mm) containing an *A. suum* egg suspension [44,45].

### 2.2. Ascaris suum Eggs

Fertilized eggs of *A. suum* were obtained from the uteri of female nematodes (*n* = 60) obtained from the intestines of pigs from organic (50%) and traditional (50%) farms. The collected eggs were centrifuged in distilled water (3× at 1000 rpm) for 3 min. *A. suum* eggs were incubated in distilled water containing 0.05% formalin, 0.05% streptomycin sulfate, and 0.01% chloramphenicol [46,47].

Experimental cultures (with the presence of entomopathogenic fungi) and control culture (without fungi) each contained a 10 mL suspension of *A. suum* eggs. *A. suum* eggs were incubated in Petri dishes (ø 50 mm) at 26 °C for 28 days.

*A. suum* eggs were observed under a light microscope (Olympus CX21, Japan; ×10; 40) on days 7, 14, 21, and 28 of incubation from a collected 0.1 mL of *A. suum* egg suspension. The following developmental stages were determined in 100 randomly observed eggs: zygote, 2–8 blastomeres, morula/blastula, gastrula, and larva. The evaluation of developmental stages was performed on 100 eggs each time and repeated twice for both experimental and control cultures.

### 2.3. Fungal Enzymatic Activity

The API-ZYM test (bioMerieux, Lyon, France) was used to semiquantitatively determine the activity of 19 hydrolytic fungal enzymes: alkaline phosphatase, esterase (C4), esterase lipase (C8), lipase (C14), leucine arylamidase, valine arylamidase, cystine arylamidase, trypsin, chymotrypsin, acid phosphatase, naphthol-AS-BI-phosphohydrolase, α-galactosidase, β-galactosidase, β-glucuronidase, α-glucosidase, β-glucosidase, N-acetyl-β-glucosoaminidase, α-mannosidase, and α-fucosidase. Assays were performed according to the manufacturer’s instructions. API-ZYM strips were incubated with mature (7 day) fungal cultures grown on PDA (transferred to sterile saline) and then incubated at 37 °C for 4 h. Hydrolytic activity was determined in nanomoles of hydrolyzed substrate on a color scale from 0–5 provided by the manufacturer, where: 0—negative reaction, 1—5 nM, 2—10 nM, 3—20 nM, 4—30 nM, and 5—40 nM, or more. 1–2 was interpreted as low, 3 as moderate, and 4–5 as high enzymatic activity.

### 2.4. Cytotoxicity of Entomopathogenic Fungi in MTT Assay

Cytotoxicity was determined using tetrazole salt MTT (3[4,5-dimethylthiazol-2-yl]-2,5-diphenyltetrazolium bromide, Sigma Aldrich, Darmstad, Germany). The study was conducted using the porcine kidney (SK) cell line. The MTT colorimetric assay for cytotoxicity is based on the conversion of yellow tetrazole salts to purple water-insoluble formazan crystals. The salt reduction takes place exclusively in the mitochondria of living and metabolically active cells. If cells are damaged or destroyed by the toxin, the salt does not transform and the yellow color of the tetrazole salts is retained [48].

The study was performed using a swine kidney (SK) cell line. The cells were cultured in MEM (Minimum Essential Medium Eagle; Sigma-Aldrich) supplemented with an antibiotic solution (stock solution: 10,000 units of penicillin and 10 mg of streptomycin per mL in 0.9% NaCl (Sigma-Aldrich)), and 5% fetal calf serum (Sigma-Aldrich) in a CO_2_-incubator (CB, BINDER GmbH, Tuttlingen, Germany) (5% CO_2_, 37 °C, 98% humidity). 

Extracts were prepared from the fungal strains grown in the Petri dishes (PDA medium). The fungal strains were transferred to a sterile stomacher bag and extracted twice with 50 mL of chloroform for 4 min in a laboratory paddle blender (BagMixer^®^ 400, Intersciences, Saint Nom la Bretêche, France). Chloroform was passed through a filter into a round bottom flask and evaporated to dryness in a vacuum evaporator. The evaporation residue was dissolved in 2 mL of chloroform using an ultrasonic bath and pipetted into the vials; then, the extracts were evaporated under a stream of nitrogen. Extracts were dissolved in 1 mL of mixture of ethanol dimethyl sulfoxide–minimum essential medium with Earle’s salts (MEM) (1.7 + 0.3 + 98, *v*/*v*/*v*) as described by Hanelt et al. [48]. Then, serial log 2 dilutions of sample extract were prepared (1–31.5 cm^2^/mL, 2–15.625 cm^2^/mL, 3–7.813 cm^2^/mL, 4–3.906 cm^2^/mL, 5–1.953 cm^2^/mL, 6–0.977 cm^2^/mL, 7–0.488 cm^2^/mL, 8–0.244 cm^2^/mL, 9–0.122 cm^2^/mL, and 10–0.061 cm^2^/mL).

For the next 48 h, the plates were incubated in a CO_2_-incubator (CB, BINDER GmbH, Tuttlingen, Germany) (5% CO_2_, 37 °C, 98% humidity); then, 20 µL of MTT solution in PBS (Merck, Darmstadt, Germany) was added to the wells and the whole set was incubated for another 4 h. After removing the liquid from the wells, 100 µL of DMSO was added as a solvent for the formazan crystals. After shaking for 5 min (Titramax 101 shaker, Heidolph, Schwabach, Germany), cytotoxicity was quantified using a microplate spectrophotometer (Ledetect 96 Microplate Reader, Labexim Products, Lengau, Austria) coupled with MikroWin 2010 OEM version (Mikrotek Laborsysteme GmbH, Overath, Germany) based on the absorbance measured at a wavelength of 510 nm, which corresponded to the maximum absorption of the formazan derivative. If the absorbance was less than 50% of the cell division activity, all samples analyzed were considered toxic.

The cytotoxicity IC 50 (sample concentration at which cell proliferation is inhibited by 50% compared to control cells) was determined on the basis of serial dilutions according to the scale of Hanelt et al. [48]. The cytotoxicity of the analyzed fungal strains was reduced by the IC50 value of the control. Semi-quantitative scale for cytotoxicity grading was adopted: the low cytotoxic effect (+) with a half maximal inhibitory concentration (IC_50_) value from 31.251 cm^2^/mL to 7.813 cm^2^/mL, the medium cytotoxic effect (++) with a value from 3.906 cm^2^/mL to 0.488 cm^2^/mL, and the high cytotoxic effect (+++) with a value from 0.244 cm^2^/mL to 0.061 cm^2^/mL. No cytotoxic effect was assessed if the extract concentration at 31.251 cm^2^/mL failed to inhibit the growth of the swine kidney cell line (SK) (and if the percentage of extinction with respect to the control group was ≥50%).

### 2.5. Statistical Analyses

Statistical analyses were performed using Statistica version 12 software (StatSoft. Inc., Hamburg Germany). Independent samples t-test was used to determine differences between the two groups (control vs. experimental). Differences at *p* < 0.05 were considered significant.

## 3. Results

### 3.1. Influence of Entomopathogenic Fungi on the Embryonic Development of A. suum

**Figure 3:**
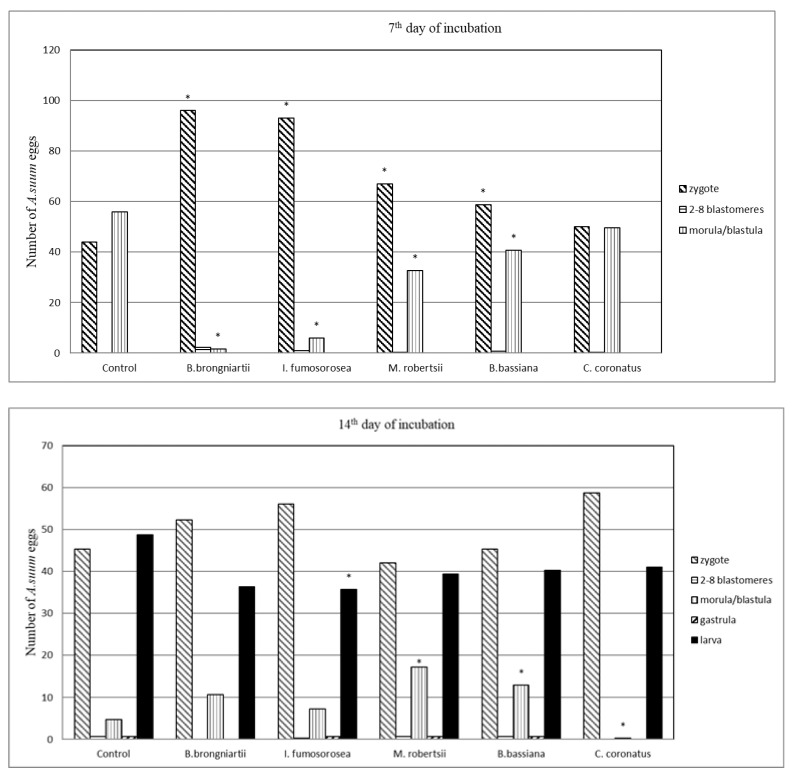

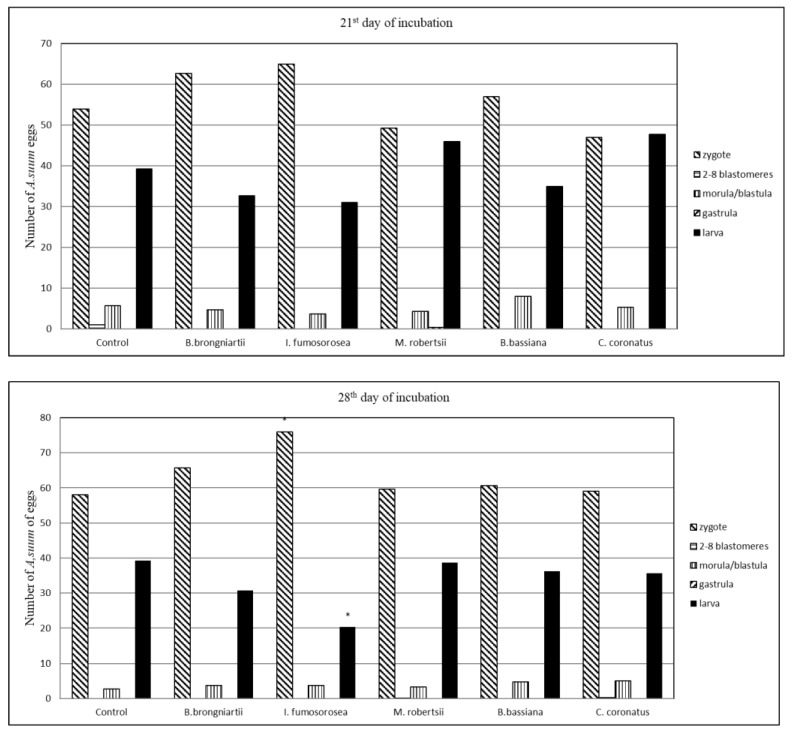
The mean number of *Ascaris suum* eggs at 7th, 14th, 21st and 28th day of incubation in control and in the presence of fungi: *Beauveria brongniartii*; *Isaria fumosorosea*; *Metarhizium robertsii*; *Beauveria bassiana*; and *Conidiobolus coronatus*; *—differences statistically significant at *p* < 0.05.

The inhibitory effect of most of the tested fungi on the embryonic development of *A. suum* was most evident up to the 14th day of incubation (*p* < 0.05). On the 7th day of incubation, the inhibitory effect on the embryogenesis of *A. suum* was shown by all tested fungal species except *C. coronatus* (Figure 3). The most inhibitory effect on the development of *A. suum* eggs—expressed by a higher percentage of zygotes compared to the control—was shown by the following fungal species, respectively: *B. brongniartii*, *I. fumosorosea*, *M. robertsii*, and *B. bassiana* (*p* < 0.05). At the same time, there was a lower percentage of *A. suum* eggs at the morula/blastula stage incubated with these fungal species (*p* < 0.05). 

On day 14 of embryogenesis, a higher percentage of eggs at the morula/blastula stage was found in eggs incubated with *M. robertsii* and *B. bassiana* compared to the control, while a lower percentage of eggs at this stage (*p* < 0.05) was found in eggs incubated with *C. coronatus*. In the presence of all fungi tested, the percentage of larvae was lower compared to the control, but statistically significant differences were found only for *I. fumosorosea*. On day 21, a higher percentage of eggs at the zygote stage was found in the presence of *B. brongniartii*, *I. fumosorosea*, and *B. bassiana*, but these differences were not statistically significant compared to the control. On the 28th day of incubation, the highest percentage of eggs at the zygote stage and the lowest percentage of eggs at the larval stage were found only in the presence of *I. fumosorosea* (*p* < 0.05). None of the five entomopathogenic fungal strains tested colonized the egg shells of *A. suum*.

### 3.2. The Enzymatic Activity of Entomopathogenic Fungi

Among the strains tested in the API-ZYM^®^ test, *I. fumosorosea* showed the largest spectrum of hydrolase production (positive reaction of 3–5; 20–40 nM or more) (13 out of 19 enzymes). All strains except *B. brongniartii* were capable of producing leucine arylamidase, while the positive reaction in the production of valine and cysteine arylamidase and trypsin was shown only by the strain *I. fumosoresea* (Table 1). All strains, except *B. brongniartii*, showed C4 esterase activity, while C8 esterase was produced only by strains *I. fumosorosea* and *C. coronatus*.

None of the strains tested was capable of synthesizing lipase (C14), chymotrypsin, α-galactosidase, α-mannosidase, β-glucuronidase and α-fucosidase. *B. brongniartii*, and *I. fumosorosea* showed β-galactosidase activity (3–5; 20–40 nM or more). All strains showed very high activity of acid phosphatase (5; 40 nM or more) and β-glucosidase (3–5; 20–40 nM or more). High activity (3–5; 20–40 nM or more) of all strains was also found in the production of naphthol phosphohydrolase and N-acetyl-β-glucosaminidase. In contrast, a very high α-glucosidase activity was found only in *I. fumosorosea* (5; 40 nM or more).

### 3.3. Cytotoxicity of Entomopathogenic Fungi in MTT Test

In the MTT test, only the strain *Metarhizium robertsii* showed the lowest IC ratio (IC_50_ 3.91 cm^2^/mL) and moderate cytotoxicity among the entomopathogenic fungi tested. For *Isaria fumosorosea*, the ratio was slightly higher (IC_50_ 7.81 cm^2^/mL) and corresponded to low cytotoxicity. A similarly low level of cytotoxicity characterized the other tested fungal species (Table 2).

## 4. Discussion

This study is part of a series of experiments on the effects of different trophic groups of fungi on *A. suum* embryogenesis, some of which have been published on soil fungi [45]. In both the studies on soil fungi and entomopathogenic fungi, there was a control group in which a surprisingly high percentage of *A. suum* eggs (more than 50%) remained in the zygote stage until the end of incubation. An explanation for this phenomenon may be that the eggs used in the study (in both the experimental and control cultures) were obtained from 60 female pigs obtained from one organic (N = 30) and one conventional (N = 30) farm. On organic farms, one method of reducing parasite infestations is the use of feed additives in the form of medicinal plants. The active constituents of these plants reduce the level of parasitic infestation and interfere with the physiological functions of the parasite [49]. In an organic farm where 50% of the adult forms of *A. suum* were obtained, a mixture of herbs such as garlic (*Allium sativum*), pumpkin (*Cucurbita pepo*), thyme (*Thymus vulgaris*), mugwort (*Artemisia vulgaris*), and fennel (*Foeniculum vulgare*) were added to the feed, with varying nematocidal effects [50,51,52,53,54]. The presence of these herb species in the host diet was also most likely responsible for the inhibition of *A. suum* embryonic development in controls [45].

In recent decades, the fight against parasitic diseases has been based mainly on chemotherapy involving repeated administration of antiparasitic drugs. Research indicating the possibility of a natural, biological reduction of *A. suum* populations is of great practical importance. The subject of this research is very topical because, in several regions of the world, pig farming has experienced an increase in the extensiveness of *A. suum* infection, which is associated, among other things, with the use of manure or slurry as fertilizer. In Norway, an increase in the extensiveness of *A. suum* infection in pigs has been associated with increased humidity in washing pens and mechanical egg transfer [15]. 

Previous results from studies of antagonistic interactions between entomopathogenic fungi and parasitic geohelminths [29,30,31,34,40,41] inspire an evaluation of the potential capabilities of fungi in the bioregulation of invasive stages of parasites, such as the nematode *A. suum*. 

This study showed varying degrees of inhibitory effects of the tested entomopathogenic fungi on the embryonic development of *A. suum*. Observation of the entire embryogenesis of *A. suum* in contact with the fungi indicates that the potential for the antagonistic effect of most of the tested strains manifested mainly in the first 14 days of incubation, while at the end of the experiment it persisted only in *I. fumosorosea*. An exponent of the nematostatic effect of *I. fumosorosea* was a lower percentage of *A. suum* larvae on the 7th, 14th, and 28th days of incubation and a higher percentage of eggs at the zygotic stage on the 7th and 28th days of incubation, compared to the control (*p* < 0.05). The mechanism of this inhibitory effect may have different sources. Many authors point to the importance of the enzymatic activity of entomopathogens in causing zoomycosis [28,55,56], and anthropomycosis [57]. The results of the studies conducted also suggest that fungal enzymes may play a major role in the inhibition of *A. suum* embryogenesis. 

In fact, it was found in the most inhibitory strain of *A. suum*—strain *I. fumosorosea*, which is associated with the highest number of enzymes detected in the API-ZYM assay (13 enzymes out of 19) and their highest activity, compared to the other fungal strains (Table 1). The most important of these enzymes are probably the peptidases (cysteine and valine arylamidase), whose activity is characterized by *I. fumosorosea*. This is confirmed by studies linking the virulence of entomopathogenic fungi to the production of the mentioned enzymes [58]. In the first phase of insect cuticle degradation, other proteases Pr1 (subtilisin-like peptidase) and Pr2 (trypsin-like peptidase) are also active [25,59]. Their presence has been demonstrated in *B. bassiana* [60,61,62], *M. anisopliae* [26], and *I. fumosorosea* [63]. Our results confirm trypsin activity only for *I. fumosorosea*, which seems to warrant a key role for this peptidase in the process of cuticle penetration. However, this does not exclude the involvement of other enzymes in the overall proteolytic activity of fungi [44]. 

Another group of enzymes important for the pathogenicity of entomopathogenic fungi are lipases and esterases. Their action in the insect epicuticula enables both the hydrolysis of lipoproteins and lipids and the adhesion of fungal spores [29]. In addition, these enzymes also affect changes in the permeability of biological membranes. In our study, all strains tested showed the highest activity of acid phosphatase and naphthol-AS-BI phosphohydrolase (concentrations above 40 nmol). As in the case of proteolytic activity, the strain *I. fumosorosea* stood out among those tested, both in the number and degree of lipase and esterase activity, as confirmed by Ali et al. [63]

Individual enzymes from the group of these hydrolases are also produced by *B. bassiana*, *C. coronatus*, and *M. robertsii* (Table 1), which is also confirmed by the studies of other authors [26,29,59,64]. Not all enzymatic activity results are so clear, which may indicate differences in strain activity. This suggestion is supported by the study of Bridge et al. [65], who tested 22 strains of *M. anisopliae* and found that each strain had its own unique enzymatic profile. In their study, *M. robertsii* produced only six enzymes with moderate to high hydrolytic capacity (above 20 and 40 nmol) (Table 1). This enzymatic activity, combined with the highest degree of cytotoxicity among the fungi tested, may have been responsible for the intensity of the antagonistic effect, which translated into the inhibition of embryogenesis of *A. suum*. Thus, in further studies, it would be advisable to identify specific toxic compounds produced by entomopathogenic fungi, since the MTT assay is quantitative and not qualitative.

## 5. Conclusions

The conducted study indicates that none of the fungal strains tested have nematocidal activity against *A. suum* eggs and do not meet the criteria required for their use in bioregulation of the parasite’s dispersal stages. However, among the strains tested, the *I. fumosorosea* and *M. robertsii* strains stood out, combining the highest metabolic activity with nematostatic activity against *A. suum*. In the perspective of further research, it is necessary to take into account the method of raising pigs (organic farms vs. traditional farms) because of the possibility of a potential effect of the host diet (i.e., the presence of different types of herbs in the feed) on the embryogenesis of *A. suum*.

## Figures and Tables

**Table 1:** The production of 19 hydrolases by entomopathogenic fungal species in the API-ZYM^®^ test.

	Enzyme	*Beauveria* *bassiana*	*Beauveria* *brongniartii*	*Conidiobolus* *coronatus*	*Isaria* *fumosorosea*	*Metarhizium* *robertsii*
1	Control	0	0	0	0	0
2	Alkaline phosphatase	1	1	3	3	0
3	Esterase (C4)	3	1	3	3	3
4	Esterase lipase (C8)	1	1	3	3	0
5	Lipase (C14)	0	0	0	0	0
6	Leucine arylamidase	3	1	3	3	3
7	Valine arylamidase	0	0	2	3	0
8	Cystine arylamidase	0	1	2	3	0
9	Trypsin	0	0	0	3	0
10	Chymotrypsin	0	0	0	1	0
11	Acid phosphatase	5	5	5	5	5
12	Naphtol-AS-BI-phosphohydrolase	3	5	5	5	5
13	α-galactosidase	0	0	0	0	0
14	β-galactosidase	1	3	2	5	0
15	β-glucuronidase	0	0	0	1	0
16	α-glucosidase	0	0	0	5	0
17	β-glucosidase	5	5	5	5	3
18	N-acetyl-β-glucosaminidase	3	5	5	4	5
19	α-mannosidase	0	0	0	0	0
20	α-fucosidase	1	1	1	0	0

0—negative reaction; 1—5 nM; 2—10 nM; 3—20 nM; 4—30 nM; and 5—40 nM or more. The 1–2 score was interpreted as a low, 3 as a moderate, and 4–5 as a high enzymatic activity.

**Table 2:** Cytotoxicity of entomopathogenic fungi in the MTT assay.

No	Entomopathogenic Fungi	Step	IC_50_ (cm^2^/mL)	Degree of Cytotoxicity
1	Control PDA	2	15.625	+
2	Control cells	-	-	-
3	*Beauveria bassiana*	1	31.25	+
4	*Beauveria brongniartii*	1	31.25	+
5	*Conidiobolus coronatus*	2	15.625	+
6	*Isaria fumosorosea*	3	7.813	+
7	*Metarhizium robertsii*	4	3.906	++

Scale of cytotoxicity: +—the low cytotoxic effect with IC_50_ value from 31.251 cm^2^/mL to 7.813 cm^2^/mL; ++—the medium cytotoxic effect with IC_50_ value from 3.906 cm^2^/mL to 0.488 cm^2^/mL.

## Data Availability

Data are contained within the article.
